# A Validated UHPLC–MS/MS Method to Quantify Eight Antibiotics in Quantitative Dried Blood Spots in Support of Pharmacokinetic Studies in Neonates

**DOI:** 10.3390/antibiotics12020199

**Published:** 2023-01-18

**Authors:** Qian Liu, Lanyu Liu, Yu Yuan, Feifan Xie

**Affiliations:** Division of Biopharmaceutics and Pharmacokinetics, Xiangya School of Pharmaceutical Sciences, Central South University, Changsha 410013, China

**Keywords:** dried blood spots, LC–MS/MS, antibiotics, pediatrics, pharmacokinetics

## Abstract

Objectives: Conduction of pharmacokinetic (PK) study in pediatric patients is challenging due to blood sampling limits. The dried blood spots (DBS) method represents a potential matrix for microsampling in support of PK studies in children. Herein, we used the Capitainer^®^ qDBS device to develop a DBS method that can collect an exact 10 µL volume of blood on a paper card. This DBS method was developed to simultaneously quantify the concentrations of eight antibiotics, including sulbactam, tazobactam, ampicillin, meropenem, cefotaxime, cefoperazone, piperacillin, and metronidazole using ultra-high performance liquid chromatography–tandem mass spectrometry (UHPLC–MS/MS). Methods: The prepared DBS samples were extracted in methanol containing acetaminophen as the internal standard at 20 °C on a block bath shaker at 500 rpm for 30 min. The extracted antibiotics were eluted on an Acquity UPLC HSS T3 column (2.1 × 50 mm, 1.8 µm) using gradient elution with a total chromatographic run time of 6.5 min. The precursor and product ions of the analytes were detected by use of the multiple reaction monitoring (MRM) mode. Results: No interfering peaks at the respective retention times of the analytes were observed in DBS samples. The lower limits of quantification (LLOQ) for the antibiotics were between 0.25 and 2.0 μg/mL, and satisfactory accuracies (intra/inter-assay bias −16.7 to +13.6%) and precisions (intra/inter-assay coefficient of variations 1.5–15.6%) were obtained for the analytes. As a proof of concept, the method was applied to DBS samples obtained from neonatal patients treated with ampicillin and piperacillin/sulbactam. Conclusions: The DBS method is simple and robust, and it can be used in children with limited blood sampling.

## 1. Introduction

Bacterial infections are a common cause of death in children under the age of five [1]. Beta-lactams and nitroimidazoles are commonly used antibiotics for the treatment of Gram-negative bacterial infections and anaerobic bacteria, respectively, in pediatrics [2,3,4]. Pediatric dosing regimens cannot be simply extrapolated from adults because of the continuing physiological and anatomical changes that occur during growth in children [5]. Appropriate dosing regimens in the pediatric population often lag the adult population, and off-label use is a frequent problem in the pediatric population, with approximately 61.5% of pediatric patients reported to be at risk for potential medication errors [6]. Determining a rational dosing regimen of antibiotics for pediatrics requires pharmacokinetic/pharmacodynamics (PK/PD) data in children. An efficient and sensitive analytical method is required to obtain the drug concentration data in plasma or blood for PK analysis.

Traditional PK sampling is usually performed by collecting venous blood via invasive venipuncture with a needle, and at least hundreds of microliters of blood should be taken for a single sample [7]. However, the advised blood sample volume (e.g., <1% of the total blood volume for a single point) and number of samples in children are limited due to the ethical issues in child protection [8]. Dried blood spot (DBS) sampling has gained considerable attention as a microsampling technique to support PK studies in pediatric populations [9]. This technique could collect microliters of blood (typically 10–30 μL) on a special filter paper in the least invasive way possible (obtained by a fingertip or heel prick). The “classical” DBS sample is usually obtained by spotting a small amount (exact volume unknown) of whole blood sample onto a filter paper, and the determination of drugs in this kind of DBS sample typically uses a subpunched-derived fixed area of the DBS sample [10]. However, the hematocrit-related issues (e.g., blood volume differences in the same area of spots) may produce significant bias for the measurements [11]. Recently, different quantitative DBS sampling strategies have been designed to cope with hematocrit-based bias. The key features of these strategies are to generate the DBS sample volumetrically, followed by an analysis of the whole DBS sample. Some representative quantitative DBS sampling products include the HemaPEN^®^ device, the Mitra^®^ microsampling device, Capitainer-B device, etc. [12,13,14,15].

Several methods for the measurement of blood concentrations of antibiotics using DBS sampling have been reported [16,17,18,19,20,21,22,23,24,25,26,27]. Most of the reported DBS methods for antibiotics used “classical” DBS sampling with a diameter of 3–8 mm punches for further analysis [16,17,18,19,20,21,22,23,24,25,26], and only one or two antibiotics (mainly beta-lactams and nitroimidazoles) were measured. To our knowledge, only one quantitative DBS sampling method using a Mitra^®^ microsampling device was reported for measuring four antibiotics [27]. In addition, most of the described assays were developed using conventional high-performance liquid chromatography (HPLC) coupled with ultraviolet (UV) detection [22,24,26], photodiode array (PDA) [25], or tandem mass spectrometry (MS/MS) [16,17,18,19,23]. The use of the HPLC–UV/PDA technique often failed to provide adequate sensitivity for DBS clinical samples due to the significant sample dilution during sample pretreatment. Utilization of HPLC–MS/MS could provide adequate sensitivity for DBS analysis, but the sample analysis may be time-consuming for the measurement of multiple compounds. The combination of high-resolution ultra-high performance liquid chromatography and tandem mass spectrometry (UHPLC–MS/MS) is a preferred option for the determination of multiple antibiotics in DBS samples by providing fast turn-around analysis time and excellent sensitivity.

The aim of this study was to develop a Capitainer^®^ qDBS-based microsampling method (10 µL volume of blood per sample) coupled with UHPLC–MS/MS for simultaneous quantification of eight commonly used antibiotics (ampicillin, meropenem, cefotaxime, cefoperazone, piperacillin, sulbactam, tazobactam, and metronidazole) in pediatric patients in support of prospective pediatric PK studies.

## 2. Patients and Methods

### 2.1. Chemicals and Reagents

Sulbactam sodium, tazobactam, meropenem trihydrate, metronidazole, ampicillin sodium, cefotaxime sodium, cefoperazone sodium, piperacillin sodium, and acetaminophen were all purchased from Macklin (Shanghai, China). ULC–MS grade acetonitrile and methanol were supplied from the Anaqua™ Chemicals Supply (Wilmington, NC, USA). ULC–MS grade formic acid and ammonium formate were purchased from Macklin (Shanghai, China). Deionized water was purified using a Hitech Smart-S ultrapure water system (Shanghai, China). Whole blood was obtained from drug-free healthy volunteers and collected in heparin sodium tubes.

### 2.2. Instrumentation

Chromatographic separation and mass spectrometric detection were achieved using an Agilent 1290 Infinity II-6470 MS/MS instrument equipped with an Acquity UPLC HSS T3 (2.1 × 50 mm, 1.8 µm) column. The mobile phase consisted of ultrapure water containing 1 mM ammonium formate with 0.1% formic acid (mobile phase A) and acetonitrile (mobile phase B). The needle wash solution was 70% methanol–water. The column temperature was maintained at 35 °C, and the injection volume was 4 µL. The mobile phase flow rate was set as 0.5 mL/min, and the total running time was 6.5 min. The gradient elution was performed as follows: 0–2 min, 0–30% B; 2–3.5 min, 30–100% B; 3.5–4 min, 100% B; 4–4.3 min, 100–0% B; 4.3–6.5 min, 0% B.

The mass spectrometric detection employed an electrospray ionization (ESI) technique with rapid positive and negative ion mode switching during the multiple reaction monitoring (MRM). The ion source parameters were set as follows: gas temperature 300 °C, gas flow 5 L/min, nebulizer 45 psi, sheath gas temp 250 °C, and sheath gas flow 11 L/min. The electronic multiplier voltage (EMV) was 200 V in positive mode and 400 V in negative mode. The MRM-related conditions (e.g., precursor ions, product ions, and collision energies) for the antibiotics and internal standard (IS, acetaminophen) are shown in Table 1.

### 2.3. Preparation of Stock Solutions, Calibration Standards, and Quality Control Samples

A mixed stock solution of sulbactam (4 mg/mL), tazobactam (4 mg/mL), meropenem (1.5 mg/mL), metronidazole (1 mg/mL), ampicillin (1 mg/mL), cefotaxime (4 mg/mL), cefoperazone (6 mg/mL), and piperacillin (8 mg/mL) was prepared in 50% methanol/water (*v*/*v*). The series of working solutions were prepared by appropriate dilution of the stock solution with 50% methanol/water. The calibration standards and quality control (QC) samples (lower limit of quantitation (LLOQ), low (L), medium (M), and high (H) QCs) were prepared by adding appropriate amount of the working solution to the whole blood. To preserve the integrity of the whole blood matrix, the calibration standards and QC samples contained 5% working solutions. The concentration details regarding the calibration standards and QC samples are displayed in Table 2. The different concentrations of the antibiotics in calibrator samples were selected based on their expected maximum and trough plasma concentrations in patients on a standard treatment regimen, as described in previous literature [28,29].

### 2.4. Sample Preparation

A drop (about 20–40 µL) of the whole blood was added to the sample well of the Capitainer^®^ qDBS sampling card (Capitainer AB, Solna, Sweden). After application of blood, the capillary channel was automatically filled with 10 μL blood, which was eventually emptied onto a paper disc. After drying at room temperature for 4 h, the DBS was stored in a sealed bag with dry silica gel, and finally, the sealed bag was stored in a freezer at −80 °C.

On the day of sample analysis, the DBS samples were taken out from the freezer, and the DBS paper discs were removed from the sampling card using a clean tweezer. The DBS paper disc was transferred to a 1.5 mL Eppendorf tube, and then 200 µL of methanol containing IS (acetaminophen, 20 ng/mL) was added. The mixture was agitated (500 rpm) at 20 °C on a block bath shaker MyBL-100CS (AS ONE, Japan) for 30 min. Next, 100 μL of the extract was mixed with 100 μL of water, and the resulting solution was transferred into a 2 mL glass HPLC vial with a polypropylene insert. A volume of 4 μL final solution was injected into the UHPLC–MS/MS system for analysis.

## 3. Method Validation

The method was validated according to the guideline on bioanalytical method validation released by European Medicines Agency (EMA) [30] and the European Bioanalysis Forum (EBF) recommendations for the validation of bioanalytical methods for dried blood spots [31].

### 3.1. Selectivity

Selectivity was assessed by analyzing the blank DBS samples and comparing the chromatograms with corresponding DBS QC samples at the LLOQ level. If the response of interferences in the blank DBS samples at the retention time of each compound is less than 20% for the analytes and 5% for the IS in the DBS QC-LLOQ samples, the interfering components are considered negligible.

### 3.2. Carryover

Carryover was evaluated by injecting a blank sample after injection of the highest concentration of the calibration standard. An acceptable carryover was deemed if the peak area of the blank sample was less than 20% of peak response for the analytes in the DBS QC-LLOQ samples and less than 5% for the IS.

### 3.3. Calibration Curve

The calibration curves of the eight antibiotics were established according to their blood concentration ranges using eight calibration standards (Table 1). The reverse calculated concentrations of the calibration standards should be within ±15% of the nominal value, and the QC-LLOQ samples should be within ±20% of the nominal value. At least 75% of the calibration standards should meet the above criteria.

### 3.4. Accuracy and Precision

Accuracy and precision were evaluated by analyzing DBS QC samples at four concentration levels (QC-LLOQ, QC-L, QC-M, and QC-H) over three analytical runs. Six replicates of the QC samples were analyzed for each concentration level during an analytical run. Within-run accuracy and precision were determined based on the data from the same analytical run (*n* = 6), and the between-run accuracy and precision were determined based on the data from three separate analytical runs (*n* = 18). Precision was calculated as the coefficient of variation (%CV), and accuracy was calculated as the relative deviation in the determined concentration of a standard from that of its nominal concentration. The determined accuracy should be within 85–115% (80–120% for the QC-LLOQ samples), and the calculated precisions should be within 15% (20% for the QC-LLOQ samples).

### 3.5. Matrix Effect

The matrix effect (ME) was evaluated at two QC concentration levels (QC-L and QC-H). The ME was evaluated both for the extract of the blank DBS paper disc (blood + paper) and blank paper disc (without blood) to identify the potential source for the observed matrix effect. The matrix factor (MF) was calculated as the ratio of the peak areas of the analytes in the extract of the blank DBS paper disc (blood + paper) or blank paper disc to the peak areas of the analytes in the pure solution at the same concentration (*n* = 6). The IS normalized matrix factor (IS-MF) was calculated by dividing the MF of the analytes by the MF of the IS.

### 3.6. Recovery

Recovery was assessed at QC-L and QC-H concentration levels with six replicates for each level. The recovery was determined by calculating the peak area ratio of the analytes in extracted QC samples and samples spiked after extraction at the same concentration.

### 3.7. Stability

Analyte stability was evaluated with QC-L and QC-H samples in three replicates. The autosampler stability (20 °C) of the processed QC samples was determined for 10 h. Freeze-thaw stability was tested for three repeated freeze-thaw cycles from −80 °C to room temperature (25 °C). Storage stability was tested at room temperature for 24 h, 14 °C for 72 h, and −80 °C for 30 days.

Stability was determined by comparing the peak areas of the analytes in DBS samples under different storage conditions against the responses of the analytes in freshly prepared DBS samples. A deviation in the peak areas of less than 15% was considered stable.

### 3.8. Application to Clinical Samples

The developed method was applied to clinical DBS samples from neonates treated with antibiotics for bacterial infections. The neonatal patients were treated with intravenous infusion of piperacillin/sulbactam or ampicillin, and the DBS samples were collected by heel pricking at 0.5 h (end of the infusion), 2 h, 4 h, and 7 h after drug administration. The prepared DBS samples were dried at room temperature for 4 h and then stored in a −80 °C refrigerator until measurement. The blood concentration of the DBS samples was determined using the procedure described in Section 2.4 “Sample Preparation”. The study protocol was approved by the institutional review board of Xiamen Maternal and Child Health Hospital, and written informed consent was obtained from the guardian.

## 4. Results and Discussion

### 4.1. Method Development

#### 4.1.1. Chromatography and Mass Spectrometric Detection

The optimal ESI ionization mode was first screened for the antibiotics and IS. Sulbactam was found to be best detected in the negative ionization mode, while other compounds achieved higher responses in the positive ionization mode. Therefore, a rapid ionization switching between positive and negative modes was employed to allow simultaneous monitoring of all analytes. The adduct ion form used for sulbactam is [M-H]^−^, and the adduct ion form for other analytes is [M+H]^+^. The product ions for each analyte are selected by tuning the collision energy to obtain the optimum ion response.

The chromatographic conditions were also systemically investigated and optimized. Considering that most of the target antibiotics are hydrophilic compounds, an Acquity UPLC HSS T3 (2.1 × 50 mm, 1.8 µm) column was selected due to its compatibility with 100% aqueous mobile phase. Initially, the aqueous mobile phase of 0.1% formic acid was evaluated, while we failed to achieve a chromatographic separation for meropenem, metronidazole, sulbactam, and ampicillin with different gradient elutions. Then, we switched to the aqueous mobile phase of 5 mM ammonium formate plus 0.1% formic acid, and under this condition, we could achieve a near baseline separation for all compounds. However, the response of sulbactam was about 10-fold lower compared with that of 0.1% formic acid aqueous mobile phase. A reduction of the concentration of ammonium formate to 1 mM increased the sulbactam response by about 4-fold. Although the sulbactam response is still lower than that of 0.1% formic acid, the achieved sensitivity is already high enough to measure sulbactam in clinical samples. As a result, 1 mM ammonium formate with 0.1% formic acid was selected as the final aqueous mobile phase.

Once the chromatographic conditions were optimized, we performed a carryover pretest for all compounds. We found that most of the compounds (except for sulbactam and IS) had different levels of carryover. Among them, the carryovers of meropenem, cefotaxime, and ampicillin were beyond 20%, with values of 102%, 31%, and 24%, respectively. Different troubleshooting efforts were then tried to eliminate or minimize the carryover. First, the blank injection (starting the chromatography run without injecting sample) after an injection of the high-concentration sample was performed, and the carryover peaks were still observed. This indicated that the sample loop and needle of the autosampler was not the source of the carryover issue. Second, the chromatography column was suspected to be the source of the carryover, and the column was replaced with a zero-dead-volume connecter. A blank run was followed after sample injection, and the carryover was again produced. Furthermore, an extensive column washing after the gradient elution also did not reduce carryover significantly. These experiments demonstrated that the carryover problem is hardware related. We then checked the flow path fittings and tubing, and a replacement of inlet stainless steel fittings and tubing for the column reduced the carryover of meropenem (from 102% to 40%) and cefotaxime (from 31% to 12%), while the carryover for ampicillin remained unchanged. The carryover for meropenem and ampicillin in the second blank injection was less than 20%, which indicates that a double blank injection is needed during the sample analysis for these two analytes.

#### 4.1.2. Extraction Conditions

**Figure 1:**
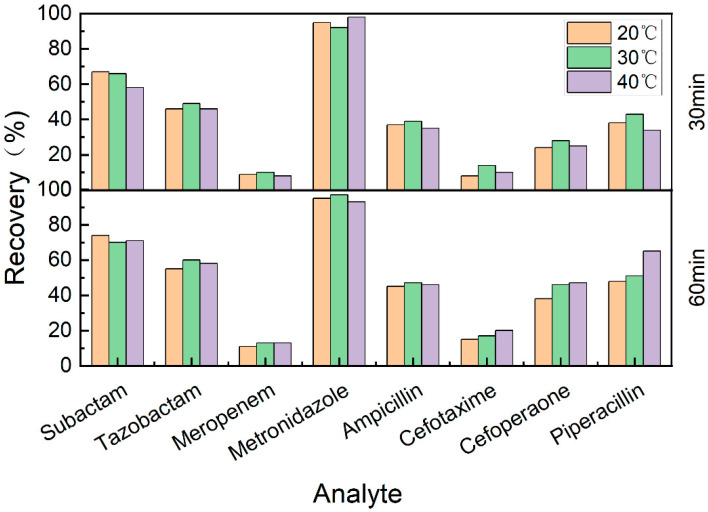
Extraction recoveries of DBS samples (QC-M level) evaluated at different conditions. The specific extraction conditions were shown as follows: 20 °C for 30 min, 20 °C for 60 min, 30 °C for 30 min, 30 °C for 60 min, 40 °C for 30 min, and 40 °C for 60 min.

The extraction recoveries of the analytes in DBS samples were evaluated at the conditions of 20 °C for 30 min, 20 °C for 60 min, 30 °C for 30 min, 30 °C for 60 min, 40 °C for 30 min, and 40 °C for 60 min with a fixed rotation speed of 500 rpm. As shown in Figure 1, most of the antibiotics (sulbactam, tazobactam, meropenem, metronidazole, and ampicillin) showed a minor difference in recoveries under different extraction conditions. Cefotaxime, cefoperazone, and piperacillin all showed the highest extraction recoveries at 40 °C for 60 min, which were significantly higher compared with the condition of 20 °C for 30 min. As these three drugs showed a high mass spectrometry response, the relatively low extraction recovery could still attain the required sensitivity for sample analysis. To reduce the extraction time and improve the sample preparation efficiency, the final extraction condition was determined to be 20 °C for 30 min.

### 4.2. Method Validation

#### 4.2.1. Selectivity

**Figure 2:**
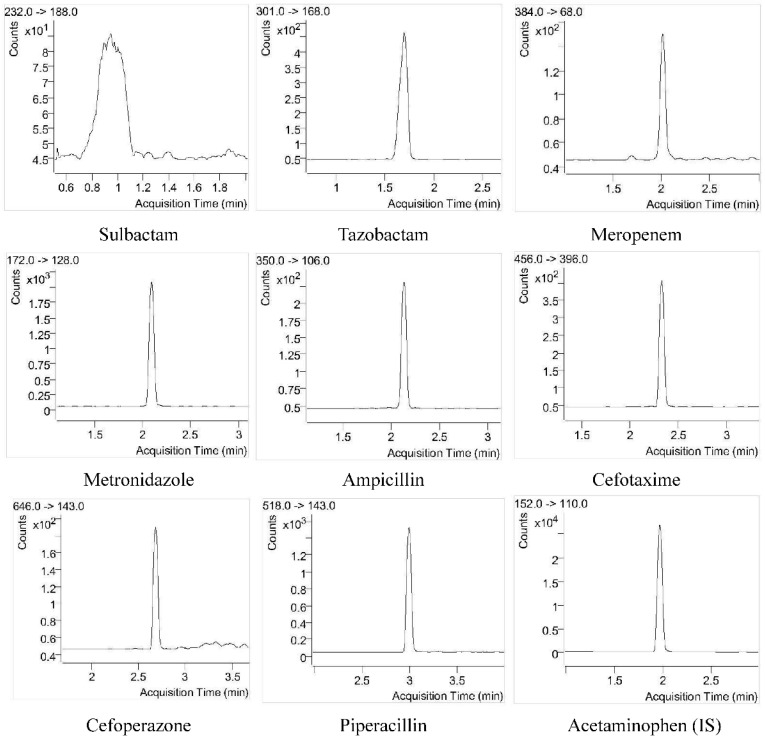
Representative chromatograms of the eight antibiotics and internal standard (IS, acetaminophen) in a DBS sample at QC-LLOQ level. The concentrations of sulbactam, tazobactam, meropenem, metronidazole, ampicillin, cefotaxime, cefoperazone, and piperacillin in the DBS sample were 1.0, 1.0, 0.375, 0.25, 0.25, 1.0, 1.5, and 2.0 μg/mL, respectively.

The responses of the interfering compounds in the blank DBS sample were all less than 20% of the antibiotic response in the DBS QC-LLOQ sample and less than 5% for the IS response. This demonstrated that the developed method is highly selective for the analytes. The representative chromatograms of antibiotics and IS in a DBS QC-LLOQ sample and blank blood DBS sample are shown in Figure 2 and Figure S1 (Supplementary data).

#### 4.2.2. Carryover

The peak responses of the analytes in the first and second blank runs after injecting the highest concentration calibration standard are shown in Supplementary Table S1. In the first blank injection, the peak areas of ampicillin and meropenem were 24% and 40% of those in the QC-LLOQ sample, and the carryovers for other antibiotics were less than 20% (less than 5% for IS). In the second blank injection, the carryovers of ampicillin and meropenem were 13% and 15%, which satisfied the acceptance criteria. Therefore, two blank injections were performed after the injection of the highest concentration sample for subsequent method validation.

#### 4.2.3. Calibration Curve

The calibration curves for sulbactam, tazobactam, meropenem, metronidazole, ampicillin, cefotaxime, cefoperazone, and piperacillin were established over the concentration ranges of 1.0–200 μg/mL, 1.0–200 μg/mL, 0.375–75 μg/mL, 0.25–50 μg/mL, 0.25–50 μg/mL, 1.0–200 μg/mL, 1.5–300 μg/mL, and 2.0–400 μg/mL, respectively. The quadratic equation model with different weighting factors or axis transformations was the best fit for the data (Supplementary Table S2). The reverse calculated concentrations of the calibration standards for all analytes were within ±15% (±20% for LLOQ samples) of the nominal values.

#### 4.2.4. Accuracy and Precision

The within-run, as well as between-run, accuracy and precision data for the eight antibiotics are shown in Table 3. The accuracies of sulbactam, tazobactam, meropenem, metronidazole, ampicillin, cefoperazone, and piperacillin all ranged from 85% to 115%, with precisions less than 15%. For cefotaxime, the accuracy of the QC-L samples was 83.3%, and the precision for the QC-M samples was 15.6%, which slightly exceeded the acceptance criteria. 

#### 4.2.5. Matrix Effect

**Figure 3:**
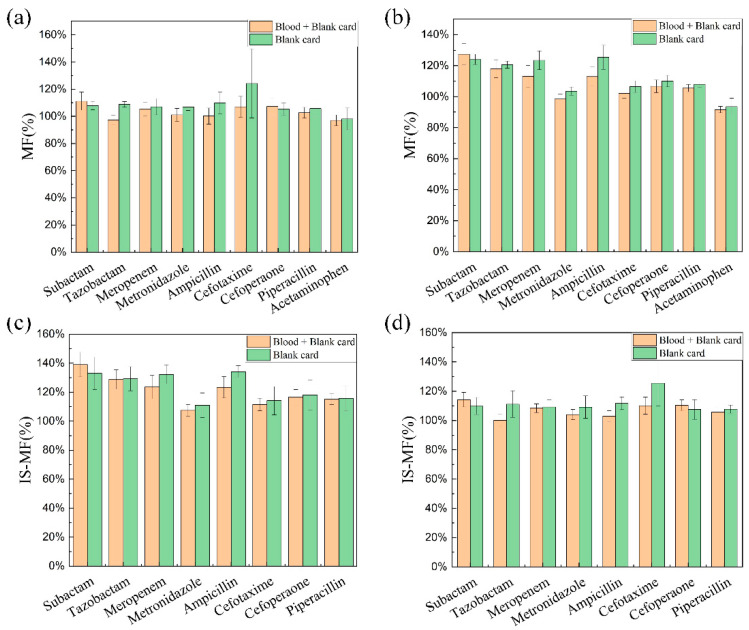
Absolute matrix factors (MF) and IS normalized matrix factors (IS-MF) for the analytes in blank paper disc and blank DBS paper disc (blood + paper). Panel (**a**), MF at QC-L concentration level; Panel (**b**), MF at QC-H concentration level; Panel (**c**), IS-MF at QC-L concentration level; Panel (**d**), IS-MF at QC-H concentration level.

The absolute MF and IS-MF of the eight antibiotics at QC-H and QC-L concentrations are shown in Figure 3. The MF and IS-MF for most of the drugs exceeded 115%, which indicates that the presence of the matrix has an enhancing effect on the ionization process of the antibiotics. Meanwhile, the MF and IS-MF of the blank paper disc and blank DBS paper disc (blood + paper) were essentially identical, meaning that the matrix source comes from the paper disc rather than blood. As declared by the provider of Capitainer^®^ qDBS, the patent blue and polyvinylalcohol membrane used for the sampling card may contribute to the matrix effects. The matrix in the sampling card is relatively consistent, and the magnitude of the matrix effect is similar for the DBS cards. Consequently, the presence of a matrix effect is not detrimental to the accuracy and precision of the assay.

#### 4.2.6. Recovery

The recoveries for the eight antibiotics at QC-L and QC-H concentration levels are shown in Supplementary Table S3. The highest recovery (96%–113%) was obtained for metronidazole, and the lowest recovery (10%–23%) was obtained for cefotaxime. The antibiotics (except for cefotaxime) showed consistent recoveries at low and high concentrations. A concentration-dependent recovery was observed for cefotaxime, and the recovery of cefotaxime at a high concentration was doubled compared to the low concentration. This may contribute to the observed bias and imprecision.

#### 4.2.7. Stability

The stability data for the QC-L and QC-H samples are provided in Table 4. High stability was observed for sulbactam, tazobactam, metronidazole, cefoperazone, and piperacillin in DBS in which no significant degradation occurred in the autosampler for 10 h, room temperature for 24 h, 14 °C for 72 h, −80 °C for 30 days, and three consecutive freeze–thaw cycles. Meropenem, ampicillin, and cefotaxime are slightly degraded when stored at room temperature for 24 h and are stable under other storage conditions.

#### 4.2.8. Method Application

**Figure 4:**
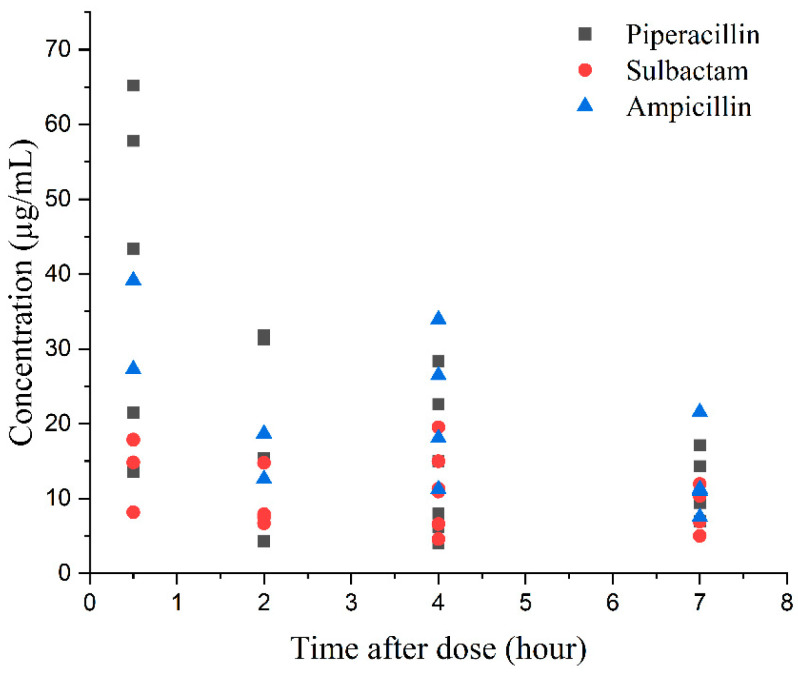
Individual concentrations versus time after dose of piperacillin, sulbactam, and ampicillin in neonatal patients with bacterial infections.

As a concluding proof-of-concept, the validated method was successfully applied to measure piperacillin, sulbactam, and ampicillin concentrations in DBS samples collected from neonatal patients with bacterial infections. Thirty-six DBS samples were obtained from fourteen neonatal patients treated with piperacillin/sulbactam or ampicillin. The individual concentrations versus time after dose are shown in Figure 4, which confirms the clinical utility of our analytical methodology. Further interpretation of the pharmacokinetic results will be performed until the completion of the clinical study.

## 5. Conclusions

We developed a rapid and sensitive DBS method for simultaneous quantification of sulbactam, tazobactam, ampicillin, meropenem, cefotaxime, cefoperazone, piperacillin, and metronidazole using UHPLC–MS/MS. The reliability of our analytical method was validated according to international guidelines. The DBS method enables a quantitative microsampling, and the sample processing is simple. As a proof of concept, this method has been applied to measure piperacillin, sulbactam, and ampicillin in clinical DBS samples from neonatal patients.

## Figures and Tables

**Table 1:** Mass spectrometric detection conditions for eight antibiotics and internal standard (acetaminophen).

Analyte	Precursor Ion (m/z)	Product Ion (m/z)	Dwell Time (ms)	Fragmentor Voltage (V)	Collision Energy (eV)	ESI Polarity
Sulbactam	232	188	50	135	8	Negative
Tazobactam	301	168	50	135	15	Positive
Meropenem	384	68	50	135	30	Positive
Metronidazole	172	128	50	135	30	Positive
Ampicillin	350	106	50	135	25	Positive
Cefotaxime	456	396	50	135	10	Positive
Cefoperazone	646	143	50	135	25	Positive
Piperacillin	518	143	50	135	25	Positive
Acetaminophen	152	110	50	135	15	Positive

**Table 2:** Detailed concentrations of calibration standards and quality concentration (QC) samples.

Analyte	Calibration Standards (μg/mL)									QC Samples (μg/mL)			
	STD8	STD7	STD6	STD5	STD4	STD3	STD2	STD1		QC-LLOQ	QC-L	QC-M	QC-H
Sulbactam	1.00	2.00	4.00	8.00	20.00	40.00	100.00	200.00		1.00	2.00	8.00	160.00
Tazobactam	1.00	2.00	4.00	8.00	20.00	40.00	100.00	200.00		1.00	2.00	8.00	160.00
Meropenem	0.375	0.75	1.50	3.00	7.50	15.00	37.50	75.00		0.375	0.75	3.00	60.00
Metronidazole	0.25	0.50	1.00	2.00	5.00	10.00	25.00	50.00		0.25	0.50	2.00	40.00
Ampicillin	0.25	0.50	1.00	2.00	5.00	10.00	25.00	50.00		0.25	0.50	2.00	160.00
Cefotaxime	1.00	2.00	4.00	8.00	20.00	40.00	100.00	200.00		1.00	2.00	8.00	240.00
Cefoperazone	1.50	3.00	6.00	12.00	30.00	60.00	150.00	300.00		1.50	3.00	12.00	240.00
Piperacillin	2.00	4.00	8.00	16.00	40.00	80.00	200.00	400.00		2.00	4.00	16.00	320.00

**Table 3:** Accuracy and precision for target analytes.

Analyte	Nominal Concentration (μg/mL)	Within-Run (*n* = 6)			Between-Run (*n* = 18)	
		Accuracy (%)	Precision (RSD%)		Accuracy (%)	Precision (RSD%)
Sulbactam	1–2–8–160	91.9–98.0–105.2–103.9	8.9–9.5–7.2–11.0		103.8–100.7–107.7–98.7	10.6–7.9–9.2–8.4
Tazobactam	1–2–8–160	103.0–100.5–105.3–99.6	2.3–8.0–7.8–8.2		103.9–102.2–113.6–100.4	5.6–7.3–10.7–5.4
Meropenem	0.375–0.75–3–60	96.9–92.8–94.8–89.9	8.7–8.0–9.3–4.0		101.4–88.6–94.4–88.5	9.2–9.3–8.6–6.0
Metronidazole	0.25–0.5–2–40	87.2–95.8–104.5–96.8	3.4–4.2–3.6–9.3		93.9–94.8–108.4–101.6	15.0–9.2–9.7–14.0
Ampicillin	0.25–0.5–2–40	99.1–95.4–100.4–92.8	1.7–10.4–8.9–8.8		101.4–94.8–99.0–92.5	8.1–9.0–10.1–6.0
Cefotaxime	1–2–8–160	102.8–83.3–102.5–105.5	5.2–10.8–15.6–10.9		103.7–83.7–97.4–97.5	15.5–7.7–11.2–11.3
Cefoperazone	1.5–3–12–240	98.3–93.6–92.4–97.9	6.3–8.8–9.1–9.3		100.0–93.2–95.1–96.4	8.1–8.0–12.2–2.5
Piperacillin	2–4–16–320	106.6–92.1–92.2–96.7	1.5–10.3–9.2–9.7		106.1–89.1–94.7–93.4	4.1–8.2–11.9–6.5

**Table 4:** Stability data for the analytes in QC-L and QC-H samples.

Analyte	Nominal Concentration (μg/mL)	Mean Percent Accuracy (%, *n* = 6)						
		Autosampler Stability (10 h)		Freeze and Thaw Stability		Storage Stability		
						25 °C 24 h	14 °C 72 h	−80 °C 30 Days
Sulbactam	2–160	102.1–101.3		112.0–103.5		86.5–98.2	94.1–105.3	87.9–96.0
Tazobactam	2–160	104.6–105.7		107.2–99.2		90.5–93.6	94.4–104.7	97.5–90.2
Meropenem	0.75–60	90.2–86.9		90.5–96.8		79.6–78.6	88.1–89.0	91.0–90.4
Metronidazole	0.5–40	92.0–103.6		108.0–98.0		103.6–111.1	90.9–104.0	89.4–100.4
Ampicillin	0.5–40	94.8–90.5		92.4–96.1		79.6–86.1	86.1–92.0	99.8–98.5
Cefotaxime	2–160	85.6–102.5		92.8–104.3		77.5–76.6	97.5–100.9	90.8–89.3
Cefoperazone	3–240	90.8–91.4		102.6–98.2		106.8–113.4	88.8–103.1	100.5–88.6
Piperacillin	4–320	90.9–86.2		105.4–98.6		86.0–96.1	89.6–98.6	96.8–92.7

## Data Availability

All the relevant data are shown in the manuscript.

## Supplementary Materials

The following supporting information can be downloaded at: https://www.mdpi.com/article/10.3390/antibiotics12020199/s1, Figure S1: Representative chromatograms of the eight antibiotics and internal standard (IS, acetaminophen) in a blank blood DBS sample; Table S1: Carryover of target analytes following first and second blank injections after an injection of the highest concentration of the calibration standard; Table S2: Details of calibration curves for target analytes; Table S3: Recoveries of target analytes in quality control (QC) samples at low (QC-L) and high (QC-H) concentrations.
