# Supplementary material for: A Validated UHPLC–MS/MS Method to Quantify Eight Antibiotics in Quantitative Dried Blood Spots in Support of Pharmacokinetic Studies in Neonates
Source: Antibiotics (Basel). 2023 Jan 18;12(2):199. doi: 10.3390/antibiotics12020199 (PMC9952362; doi:10.3390/antibiotics12020199)
Supplement: Supplementary file 1 [file antibiotics-12-00199-s001.zip › antibiotics-2140691-supplementary.pdf]

Supplementary data

Table S1. Carryover of target analytes following first and second blank injections after an injection of the highest concentration of the calibration standard.

Analyte	Carryover (%)	
	First injection	Second injection
Ampicillin	24	13
Cefoperazone	10	2
Cefotaxime	12	5
Meropenem	40	15
Metronidazole	3	2
Piperacillin	5	2
Sulbactam	0	0
Tazobactam	4	2
Acetaminophen	0	0

Table S2. Details of calibration curves for target analytes.

Antibiotics	Axis transformation	Calibration curve	Weight factor	Regression coefficient (R^2)
Sulbactam	None	Quadratic equation	$1/X^2$	0.9937
Tazobactam	None	Quadratic equation	$1/X^2$	0.9947
Meropenem	None	Quadratic equation	$1/X^2$	0.9926
Metronidazole	Log	Quadratic equation	1/X	0.9959
Ampicillin	None	Quadratic equation	$1/X^2$	0.9932
Cefotaxime	None	Quadratic equation	$1/X^2$	0.9912
Cefoperazone	None	Quadratic equation	$1/X^2$	0.9849
Piperacillin	None	Quadratic equation	$1/X^2$	0.9836

Table S3. Recoveries of target analytes in quality control (QC) samples at low (QC-L) and high (QC-H) concentrations.

Analyte	Mean recoveries (%)	
	QC-L (n=6)	QC-H (n=6)
Sulbactam	76.2	83.8
Tazobactam	55.9	54.9
Meropenem	12.3	10.7
Metronidazole	95.9	112.5
Ampicillin	54.7	46.1
Cefotaxime	10.0	22.9
Cefoperazone	30.9	33.1
Piperacillin	51.5	67.2

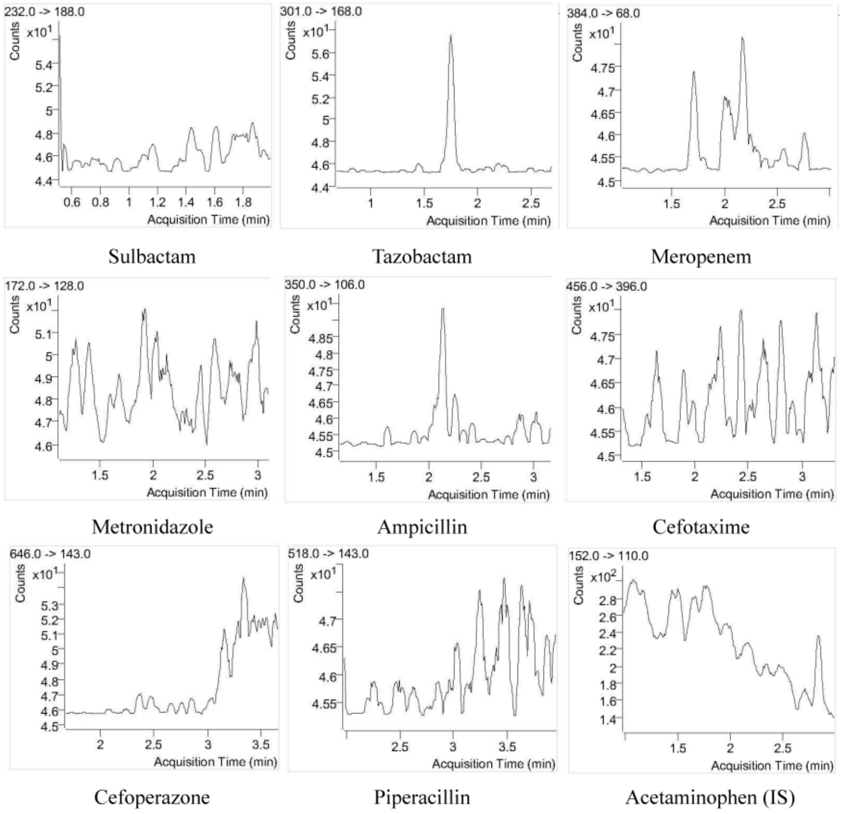

Figure S1. Representative chromatograms of the eight antibiotics and internal standard (IS, acetaminophen) in a blank blood DBS sample.
